# The Moderating Effect of Organizational Identification on the Relationship Between Organizational Role Stress and Job Satisfaction

**DOI:** 10.3389/fpsyg.2022.892983

**Published:** 2022-07-13

**Authors:** Abdullah Eriş, Özgür Kökalan

**Affiliations:** ^1^Graduate Education Institute, Istanbul Sabahattin Zaim University, Istanbul, Turkey; ^2^Department of Business Administration, Faculty of Business and Management Sciences, Istanbul Sabahattin Zaim University, Istanbul, Turkey

**Keywords:** organizational identification, organizational role stress, job satisfaction, moderating analysis, Turkey

## Abstract

This study aims to investigate the moderating effect of the organizational identification level of the employees on the relationship between their organizational role stress level and job satisfaction. Data were gathered from 460 white-collar employees with snowball sampling. Covariance-based structural equation modeling (CB-SEM) was used in data analysis. According to the research results, it was found that there is a significant negative relationship between the organizational role stress level of the employees and their job satisfaction (*r* = −0.486; *p* < 0.01). This research also shows that organizational identification has a moderating effect on the negative relationship between organizational role stress and job satisfaction. An employee who has a high level of organizational identification has more job satisfaction than an employee who has less organizational identification.

## Introduction

Since individuals cannot achieve the determined goals alone, they establish organizations and work in cooperation and coordination to move the organization to the future. Responsibilities and roles of employees are determined by the specialization and division of labor, which is in line with the structure of the organization. When the role expectations demanded from the employees and the wishes of the employees do not match, role conflict occurs and as a result, the conflict causes role stress. Role ambiguity is automatically experienced when there is a conflict between the knowledge and experience of the employees regarding the role and the knowledge and experience required by the role (Ceylan and Ulutürk, 2006).

Role conflict and role ambiguity cause role stress during an ongoing period and this affects some important organizational outcomes. When the studies on the results and antecedents of role stress are examined, it is understood that the results of the studies are uncertain and show some contradictions. It is noteworthy that the relationship between the variables could not be clearly demonstrated and the cause-effect relationship could not be explained (Chang and Hancock, 2003).

The concept of organizational identification, as the expression suggests, refers to a situation that expresses unity, synchronicity, concentricity, and synchronization. In this way, it is a concept that describes positive emotions, attitudes, and behaviors such as belonging, which is formed by the association of the organization and the members thereof. The concept of organizational identification is based on the identity theory between the individual and the group. In cases where organizational identification is high, members of the organization reveal the characteristics of their organizational identities while defining themselves. In addition, they carry these identifications beyond their professional lives to their social lives. Organizational identification is foreseen as a situation that directly affects organizational performance. When the literature is examined, although there are studies that found significant relationships among the variables of organizational role stress, job satisfaction, and organizational identification, these studies were carried out on only two variables. In the literature, a model created with these three concepts has not been found. This study aims to reveal the relationships among these three variables with the model established with role stress, job satisfaction, and organizational identification. Therefore, this study is the first to be studied in the field and has a unique quality. In this study, it was investigated how an employee's organizational identification level has a moderating effect on the relationship between the role stress experienced and job satisfaction. In addition to contributing to the literature, the findings to be obtained from this study are aimed to get results that can guide the organizations that are essential parts to the subject.

Although the importance of role stress in organizational management is unquestionably accepted in studies, there are differences in the sub-dimensions of role stress. These studies seem to focus on role conflict and role ambiguity. Areas other than these sub-dimensions have been relatively less explored. Role overload is one of these sub-dimensions. Cooper and Marshall (1976) and Jung and Yoon (2013) defined role overload as a quantitative factor. On the one hand, role conflict and role ambiguity are qualitative factors. Kelly and Barrett (2011), on the other hand, argued that role overload is a subjective and quantitative factor. In addition, it is seen that the concepts of role overload and role conflict are used interchangeably in the literature (Hecht, 2001).

In most of the studies on role stress, it is seen that only the role conflict and role ambiguity sub-dimensions of role stress are examined (Behrman and Perreault, 1984; Ceylan and Ulutürk, 2006; Akar and Yildirim, 2008; Kurt, 2010; Çekmecelioglu and Günsel, 2011; Demirci and Seçilmiş, 2020). In addition, role conflict and role ambiguity and other variables studied are intention to leave, job satisfaction, performance, and job attitudes. Also, role conflict is the result of various expectations imposed on the individual from many sources (Qazi and Nazneen, 2016).

In contrast, different sub-dimensions of role stress are also encountered in the literature. Role conflict and role ambiguity, as well as other sub-dimensions studied, are role incompatibility and role overload (Conley and Woosley, 2000; Chang and Hancock, 2003; Culbreth et al., 2005). Other variables in these studies appear to be high-level needs, job results, and job satisfaction.

In the literature, it was seen that certain titles about the sub-dimensions of role stress came to the fore and generally accepted holistic studies were limited. For this reason, it is thought that scales and studies in which the sub-dimensions of role stress can be seen as a whole will contribute greatly to the literature.

In this study, first of all, the variables were explained theoretically in the theoretical framework, their relations with each other were explained and research hypotheses were formed. Then, the method and sample of the study were reported, and the results of the analyses were interpreted and the results were obtained by discussing within the scope of the relevant literature.

## Theoretical Framework

### Organizational Role Stress and Its Sub-dimensions

Employees who are under stress see every element that happens around them as a potential danger and give both physical and emotional reactions to these situations. Employees experiencing role stress experience some deviations in both their physical and cognitive functioning as a result of the pressure. The stress in business life is more than the stress in daily life and it affects the individual at a very significant level (Yeşiltaş and Ayaz, 2019).

As the level of stress increases, employees who experience appetite problems, dizziness, depression, anxiety, sugar spikes, etc. are starting to experience some health problems. With role stress, employee attendance rates decrease, injuries and accidents occur as a result of carelessness, and as a result, both individuals and organizations are negatively affected in many ways (Papoutsis et al., 2014).

Apart from individuals' own personalities, some factors, such as the long working hours in the organization, the compelling working conditions, the mismatch between the culture of the organization and the culture of the employee, and the coercive policies of the organization, can significantly increase the stress level due to the structure of the organization and the work they do, which are the leading factors of both work-related and organizational stress. In addition, problems in the private life of the employee, experiencing financial problems and family problems constitute other stress factors brought from outside of business life (Waight and Madera, 2011). It is possible to list the sub-dimensions of role stress as follows (Papoutsis et al., 2014):

- *Inter-Role Distance (IRD):* There is a conflict between the organizational role and other roles, for example, an employee's inability to manage his time between the demands of the job and the demands of the family.
- *Role Stagnation (RS):* Lack of opportunities and opportunities for learning and growth in the organization.
- *Role Expectations Conflict (REC):* Conflicting demands on the role by different citizens in the organization.
- *Role Erosion (RE):* It occurs when the task given to one employee is given to another employee later on. In this way, the employee feels that his work is not compelling.
- *Role Overload (RO):* The role and responsibilities expected of the employee exceed and the responsibilities are quite challenging.
- *Role Isolation (RI):* A situation where an appropriate link is needed between the role of one employee and the roles of other employees.
- *Personal Inadequacy (PI):* Employees' lack of knowledge, skills, and abilities required for the role to be effective.
- Self*-Role Distance (SRD):* It is the distance between the individual's values and self-concepts and the organizational role.
- *Role Ambiguity (RA):* When a person does not have a clear picture of their work goals, they struggle between the expectations of their colleagues and the scope of their job and their day-to-day work. As a result of role ambiguity, the mood of the employees deteriorates, their self-esteem decreases, their motivation to work decreases and they have the intention to leave the job.
- *Resource Inadequacy (RI):* It is experienced when the necessary capital is not available for effective role performance.

Kahn et al. (1964) conducted a preliminary study of the concept of role stress. In this study, they stated that role stress basically arises from the relationship between the sender and receiver in communication. Accordingly, the factors affecting role stress are organizational structures, the job itself, and the characteristics of individuals. In classical organizational structures, it is essential to define precisely the duties, authorities, and responsibilities. However, it is seen that organizational activities are not so static and static in reality. Therefore, roles diversify and differentiate for alternative or compelling reasons (Kahn et al., 1964; Wen et al., 2020).

Role stress is manifested by the weakening of the sense of satisfaction of the purpose, desire, and future expectations of individuals, which depend on many variables, and as a result of losses in performance. In summary, role stress, which arises as a result of role conflict, arises from organizational and individual conflict areas (Rizzo et al., 1970).

Rizzo et al. (1970) revealed that role ambiguity and role conflict are factorial definable and independent of the structure.

Role stress refers to situations in which role obligations are unclear, uncomfortable, difficult, contradictory, or impossible. It states that role stress is the lack of information that includes rules, duties, and responsibilities related to one's job and how to perform it. Role stress is a common problem in organizations. As individuals may have different expectations about what their roles are and what they actually do, they stated that the most important sources of stress in an organization are role conflict and role ambiguity. Role conflict is defined as contradictory role expectations from the members of the organization, while role ambiguity is expressed as situations where the person does not have the necessary information about their roles or cannot fully understand their role (Demirci and Seçilmiş, 2020).

Role stress is one of the sources of job stress for employees, and role stress has two main components, namely, role ambiguity and role conflict. Role ambiguity is one of the most common role stressors for many employees. Role ambiguity refers to the situation where there is no clarity about the roles assigned to individuals. Role ambiguity occurs when the expectations of the organization from a job and the expectations of customers and people outside the organization differ, or when there is a lack of information about the job and task, opportunities for advancement, responsibilities, and the expectations of the individual in a hierarchical position (Dogan et al., 2020).

Each role stressor results from a particular type of problem that the person playing the role faces while performing their role. There are differences between the levels of management in what type of management duties they perform and the roles they play in their jobs.

Studies have shown that there are five types of role conflicts, namely, conflict due to the role sent, role conflict between senders, conflict between roles, person-role conflict, and role-out. Conflict due to the assigned role occurs when a role sender sends roles or role-related tasks to employees that may be inconsistent with each other. Role conflict between senders occurs when the role behavior requested by a role group member is incompatible with the behavior requested by another role group member or when the role expectations from the role sender differ. Inter-role conflict occurs when an individual tries to fulfill two or more roles whose expectations are incompatible and feels pressure while trying to fulfill the requirements of these different roles. Person-role conflict arises when there is a mismatch between the expectations of the role set and the individual himself (attitudes, values, and professional behavior) who will fulfill these roles. Finally, role overload occurs when a person simultaneously fulfills more than one role and does not have the resources (time, energy, etc.) to perform them (Dogan et al., 2020).

### Organizational Identification

Organizational identification is one of the topics which has been researched for a long time in the social sciences literature. The concept of organizational identification is effective both on organizational behavior and on the level of satisfaction of the individual. As a result, it has a great impact on the effectiveness of the organization (Ashforth and Mael, 1989).

Identification is a psychological state in which a person considers himself/herself as part of a whole and sees and shows his/her behavior and attitude in a sense of belonging. Individuals experiencing identification establish and maintain a meaningful relationship with other individuals or group/s. Individuals adopt the beliefs of the organization they employ, and after a while consciously or consciously they behave in this direction. The factors that define the institution start to define individuals within the organization. In other words, as a result of organizational identity, social identities are shaped (Schaufeli and Bakker, 2010).

For an individual to identify with an organization in a meaningful way, he/she needs to be positioned in his/her minds in a more memorable and different way than other organizations. Identification occurs when members of the organization find the identity of the organization attractive. The higher the organization's image is felt by the members of the organization, the higher the level of identification occurs. It is important to have a common purpose, interaction, and a common story between the organization and the individual for the identification process. Organizational identification occurs when the identity of the organization and the values of the employees are compatible. When individuals identify themselves with the organization in organizations, the level of job satisfaction increases, motivation increases, and the rate of turnover decreases (Zhou et al., 2016).

Organizational identification is examined in the literature with many different variables. It is frequently stated in the studies that organizational identification is directly proportional to positive work results (He and Brown, 2013).

### Job Satisfaction and Its Dimension

Job satisfaction is a personal evaluation process, and it is the making of a judgment by analyzing all the conditions of the job or the results obtained as a result of the job. Consciously or unconsciously, employees react to work and exhibit their behaviors and attitudes in this direction. The concept of job satisfaction is mostly used as a psychological variable in organizations. If the employees have positive feelings while evaluating their work, it means they are experiencing job satisfaction (Ambrose et al., 2014).

Since job satisfaction is not objective and visible, it can only be expressed meaningfully with predetermined attitude criteria. The more the expectations of the employees are met, the higher the level of job satisfaction will be. Being in harmony with colleagues, organization and management staff, appropriate management style, satisfactory salary, and promotion opportunities are among the factors that satisfy employees in organizations (Zhou et al., 2016).

Studies show that there is a significant relationship between job satisfaction and altruism and that employees with job satisfaction help their colleagues more and show altruistic behaviors. In addition, there is a significant and positive relationship between organizational commitment and job satisfaction. Job satisfaction level of burnout individuals is low and there is a negative relationship between burnout and job satisfaction (Yang et al., 2017).

Two sub-dimensions of job satisfaction are called as external and internal satisfaction. The external satisfaction includes elements such as occupational health and safety, promotion opportunities, and wages. The internal dimension includes elements such as performance, the individual's own qualities, and relations with management. For individuals to be satisfied with the job, they should be appreciated by their managers and receive additional payments, work conditions should be reasonable, the nature of the work should match the nature of the employee, and there should be promotion opportunities. In an organization that does not have these factors, the employee will definitely have the intention to leave the job after a certain period of time (Kara, 2010).

For employees to experience a sense of job satisfaction, the job itself should be suitable for the person, the work environment should be suitable for the person, and the employees should participate in the decisions made in the organization. The factors such as availability of training opportunities, the satisfaction of the wages, the satisfaction of the colleagues, the opportunities for promotion, the awarding, the appreciation, and the organizational commitment support are important (Gürsoy et al., 2019).

### Role Stress and Job Satisfaction

Role stress has been the subject of research in the literature as it directly affects the productivity of organizations. At the same time, it is known that the high role stress harms the psychological health of the employees first and then their biological health (Ambrose et al., 2014). Studies show that employees who experience role stress are unfaithful to the organization, are in search of a job, are not satisfied with their job, have a feeling of burnout, are absent from work, and either compulsorily or voluntarily leave the job. Studies show that the level of job satisfaction of the employees of the organization with high role stress is extremely low (Ertürk, 2010).

High workload in organizations, and conflicts between employees and managers cause role stress. This situation directly leads to a decrease in the level of job satisfaction and thereby results in the intention of employees to leave the job. It has been proved as a result of this research that the support of the employees by their colleagues and managers reduces stress and increases job satisfaction (Börk and Adigüzel, 2015).

*H*_1_*: Organizational role stress is negatively related to job satisfaction*.

### Organizational Identification on Job Satisfaction

Human resources are the most important element for organizations to leave their competitors behind and reach their goals. If the management of the organization ensures that the employees identify with the organization and connect to the organization with a sense of loyalty and belonging, the employees are more satisfied with the work they do (Akova et al., 2015). This situation brings organizational success and high organizational performance. Organizations that act with the awareness that the behavior of their employees directly affects the success of the organization continue to maintain their presence in the market (Bayram, 2019).

Studies in the literature show that there is a significant and positive relationship between organizational identification and job satisfaction. Employees who identify with the organization are more committed to their jobs and continue their roles in job satisfaction (Akbolat et al., 2011).

**Figure 1:**
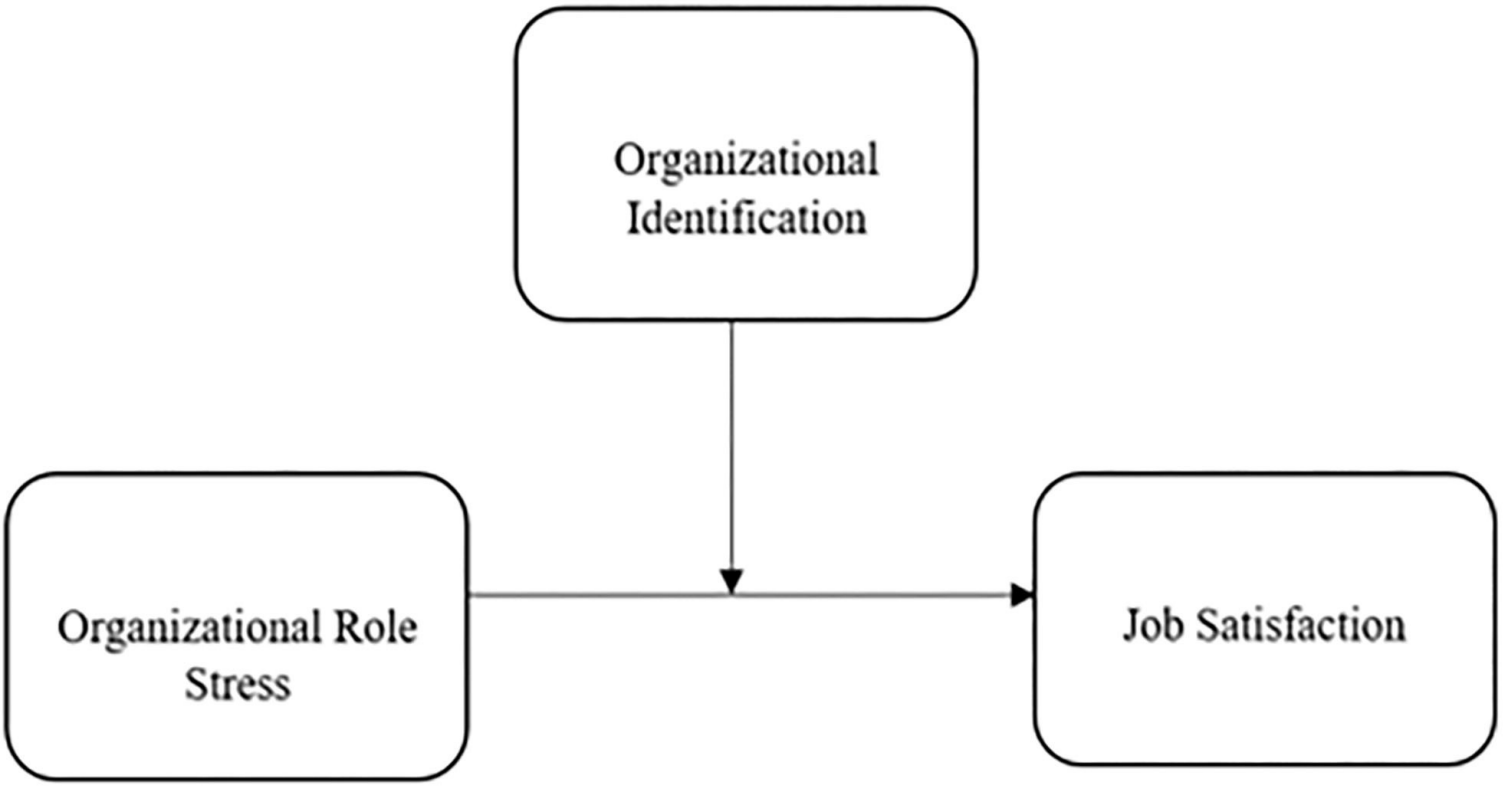
Research model.

In the literature, although there are studies explaining the relationship between organizational identification and job satisfaction, there is no study that examines the moderating effect of organizational identification on the relationship between organizational role stress and job satisfaction. With this research, it is aimed to fill this gap in the field. It is thought that the results of research will make important contributions especially to the field of organizational behavior. The second hypothesis constructed within the scope of the moderating analysis and research model is given as follows:

*H*_2_*: Organizational identification moderates the relationship between organizational role stress on job satisfaction, such that an employee who has high organizational identification has more job satisfaction than an employee who has less organizational identification*. (Figure 1).

## Methodology

### Sampling and Procedure

Data were gathered from white collar employees working in different service and manufacturing companies in Turkey during September–December 2021. In this study, the questionnaire was prepared by Google Form and distributed to participants *via* email. In the data collection process, Snowball sampling was also used to increase the number of participants. The Google Form's link was shared by some participants with their friends. In this study, 460 questionnaires were collected. Consent was obtained from all participants before the study. In the beginning of the forms sent to the participants, it was declared that no information about the participants and any data obtained would be shared with third parties. This information has also been added to the text.

Data were collected at three different times over 4 months with 1-month lags to reduce the common method bias and avoid biasing effects of occasional factors (Podsakoff et al., 2012; Matthews et al., 2014). The questionnaire used within the study consisted of four parts. In the first part, questions were asked to determine the basic socio-demographic characteristics of the participants. In the next section, questions about the organizational role stress scale were included. In the third part, questions were included to measure the organizational identification levels of the participants. With the questions in the final part, it was tried to measure the job satisfaction levels of the participants.

When the socio-demographic status of 460 white-collar employees participating in this research was examined, it was seen that 275 (59.8%) work in the service sector and 185 (40.2%) work in the manufacturing sector and 129 (28%) are women and 331 (72%) are men. It was also seen that the average age of the participants is 34.2; ≈60% of them are married and 72% of them are at least university graduates. It was determined that 52% of the participants define their income as medium level and they have an average of 10.25 years of working experience.

### Data Collection Tools

“Organizational Role Stress Scale,” “Organizational Identification Scale,” and “Job Satisfaction Scale” were used in this study.

This study used the “Organizational Role Stress Scale (ORS)” of Pareek (1993). A total of 50 items in the questionnaire assess “Inter-Role Distance (IRD),” “Role Stagnation (RS),” “Role Expectations Conflict (REC),” “Role Erosion (RE),” “Role Overload (RO),” “Role Isolation (RI),” “Personal Inadequacy (PI),” “Self-Role Distance (SRD),” “Role Ambiguity (RA),” and “Resource Inadequacy (RI)” dimensions. The scale was scored using the same 5-point Likert, ranging from 1 (if you never or rarely feel this way) to 5 (if you frequently feel this way). Sample items are “My role tend to interfere with my family life” and “My work load is too heavily.” The sum of all item scores was considered as the total organizational role stress score, and a higher score reflected a higher level of organizational role stress. As a result of confirmatory factor analysis performed to the scale, six questions were eliminated. After the elimination process, the fit indexes were as follows, *x*^2^/df = 4.113, GFI = 0.911, AGFI = 0.883, CFI = 0.908, and RMSEA = 0.089. Therefore, the model fit was determined as the acceptable level (Hair et al., 1998). The Cronbach's alpha score (CAS) for OJS was 0.866.

“Organizational Identification Scale (OIS)” was designed by Ashforth and Mael (1989), which includes 6 items covering one dimension called as “Cognitive Identification.” Sample items are “I am very interested in what others think about my company” and “This company's successes are my successes. Responses followed a 5-point Likert format (1 = Strongly agree, 5 = Strongly disagree). The sum of all item scores was considered as the total organizational identification score, and a higher score was evaluated as a higher level of organizational identification. As a result of confirmatory factor analysis, the fit indexes were as follows, *x*^2^/df = 3.814, GFI = 0.920, AGFI = 0.907, CFI = 0.924, and RMSEA = 0.076. Therefore, the model fit was determined as a very good level (Hair et al., 1998). The CAS for OIS was 0.865.

“Minnesota Job Satisfaction Scale (JSS)” was designed by Weiss et al. (1967). It consisted of 20 items and two dimensions called as “Intrinsic Satisfaction” and “Extrinsic Satisfaction.” Sample items are “Being able to keep busy all the time,” and “The chance to try my own methods of doing the job.” Responses followed a 5-point Likert format (1 = very unsatisfied; 5 = very satisfied). The sum of 20 item scores was considered as the total job satisfaction, and a higher score was evaluated as a higher level of job satisfaction. As a result of confirmatory factor analysis, the fit indexes were as follows, *x*^2^/df = 3.714, GFI = 0.904, AGFI = 0.886, CFI = 0.911, and RMSEA = 0.084. Therefore, the model fit was determined as the acceptable level (Hair et al., 1998). The CAS for JSS was 0.890.

## Results

### Descriptive Statistics

The data in this study were gathered from the respondents with the questionnaire. Because of this reason, common method bias should be controlled. In this process, common method bias was controlled by Harman's single-factor test. According to the test result, it was determined that the first factor explained only 32.11% of the total variance. In other words, this research did not have common method problems.

The descriptive statistics such as mean, standard deviation, and correlations about variables were analyzed and the results are given in Table 1.

**Table 1:** Basic statistics about the variables.

	**Mean**	**SD**	**Skewness**	**Kurtosis**	**AVE**	**MSV**	**CR**	**1**	**2**	**3**
Organizational role stress (ORS)	2.36	0.717	0.211	−0.769	0.56	0.45	0.76	1		
Organizational identification (OI)	3.59	1.123	−0.418	−0.780	0.52	0.46	0.76	−0.302^**^	1	
Job satisfaction (JS)	3.36	0.561	0.173	−0.152	0.54	0.47	0.79	−0.486^**^	0.471^**^	1
Gender	1.72	0.450						−0.135^**^	0.201^**^	0.177^**^
Age	34.2	8.126						−0.290^**^	0.281^**^	0.267^**^
Education level	2.80	0.739						−0.283^**^	0.213^**^	0.223^**^
8. Job experience	10.2	2.316						−0.227^**^	0.230^**^	0.196^**^

*N = 460*.

** *p < 0.01 level*.

The average scores on the ORS, OI, and JS were 2.36 ± 0.717, 3.59 ± 1.123, and 3.36 ± 0.561, respectively. According to these results, it can be said that the ORS level of the participants is low, and the OI and JS levels are moderate. When the skewness and kurtosis scores of the scales used in this research were examined, the skewness and kurtosis values of all variables were in the range of ±1.96. It was determined that the variables have a normal distribution (Meydan and Seşen, 2015). According to the correlation analysis, it was determined that ORS is negatively correlated with OI and JS (*r* = −0.302; *p* < 0.01; *r* = −0.486; *p* < 0.01). It was also seen that there is a positive correlation between OI and JS (*r* = 0.471; *p* < 0.01).

### Hypotheses Testing

The hypotheses were examined by Covariance-based structural equation modeling (CB-SEM). In the first step in CB-SEM, the kurtosis and skewness of the variables were controlled to evaluate the normality. It was seen that all data used in the study were normally distributed (Meydan and Seşen, 2015).

Under CB-SEM, two hypotheses were tested in this study. H_1_ states that ORS has a negative effect on JS. Table 2 shows that there is a significantly negative relationship between ORS and JS (β = −0.426, SE = 0.044, *t* = −9.778, and *p* < 0.01). H_1_ is supported.

**Table 2:** OI moderating analysis.

	**Job Satisfaction**			
	**H**_**1**_		**H**_**2**_	
	**β**	**SE**	**β**	**SE**
Constant	−0.995^**^	0.280	−0.559^*^	0.265
Gender	0.188	0.097	0.137	0.087
Marital Status	0.101	0.096	0.036	0.088
Age	0.071	0.049	0.011	0.046
Education Level	0.096	0.058	0.042	0.0.54
Job Experiences	−0.019	0.061	−0.010	0.056
Sector	0.060	0.084	0.057	0.077
Organizational Risk Stress (ORS)	−0.426^**^	0.044	−0.376^**^	0.041
Organizational Identification			0.343^**^	0.040
ORS* OI			0.145^**^	0.034
*R* ^2^	0.268		0.385	
*F*	23.611^**^		31.347^**^	

** *p < 0.01 level*.

* *p < 0.05 level*.

**Figure 2:**
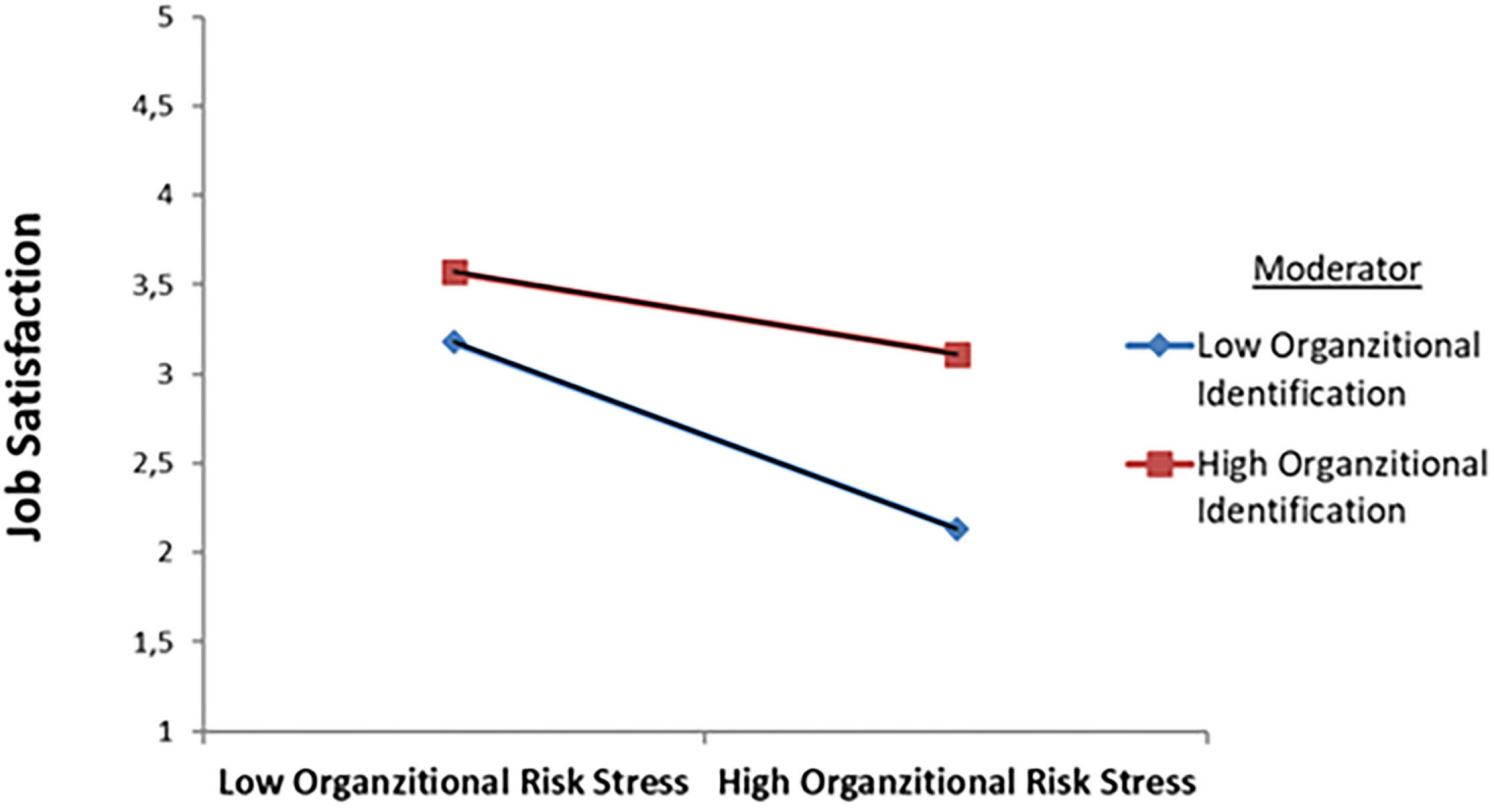
The interaction between OI and ORS.

H_2_ states that OI moderates the relationship between ORS on JS. An employee who has a high level of OI has more JS than an employee who has less OI. Table 2 shows that the OI-ORS interaction was significant (β = 0.145, SE = 0.034, *t* = 4.433, and *p* < 0.01). According to the result, OI suppresses the negative relationship between ORS and JS. Figure 2 shows this relationship. H_2_ is also supported.

## Discussion and Conclusion

This study was carried out to find the moderator effect of organizational identification on the relationship between organizational role stress and job satisfaction. As a result of the analysis, it has been determined that there is a significant and negative relationship between the organizational role stress of the employees and their job satisfaction. This result is supported by the results of many previous studies in the literature (Jamal and Badawi, 1995; Yousef, 2000; Kumar et al., 2015; Wu et al., 2019). In this research, it has also determined that the organizational identification levels of the employees have a moderating effect between organizational role stress and job satisfaction. According to the results, it has been found that an employee with high organizational identification levels has more job satisfaction than an employee who has less organizational identification. In the literature, there are studies investigating the moderator effect of organizational identification on various organizational outputs such as organizational commitment and organizational silence (Akar and Yildirim, 2008). In these studies, the positive effects of organizational identification on organizational outcomes were determined. In this study, it has been determined that the organizational identification of the employees positively affects their job satisfaction, which is an important organizational output, even if they experience organizational role stress. This result has made an important contribution to the field of organizational psychology.

These results will be beneficial for researchers and managers to carry out studies to reduce role stress and increase organizational identification in order to increase job satisfaction for employees. It is known that organizational identification can be seen as identification with the group or the whole organization. For these purposes, detailed analyzes in areas such as the past roles and psychology of the employees who are planned to join the organizations will enable them to adapt to the existing associations. In addition, providing opportunities for the human resources of the organization, especially increasing financial rewards and promotions, will be considered as tools to increase organizational identification. It should also be emphasized that career opportunities will be better than other organizations. The fact that the working groups within the organization have privileges attracts the attention of other groups. The fact that different groups work together causes the employees to highlight the characteristics of their groups. Thus, the concept of “Me” becomes the concept of “We.” In contrast, the high corporate image of the organizations can be used as another factor that positively affects the organizational identification of the employees. In short, policies should be determined to ensure organizational identification, which makes positive contributions to the organization, within the organization.

It has been observed that role stress has been investigated in the literature through its relationship with different variables. In future studies, studies can be conducted on the moderator roles of organizational identification with variables such as role stress and organizational commitment, organizational citizenship, burnout, and job performance. In this way, this method, which has been partially studied less, can contribute to the literature and interested parties.

One of the limitations of the study is that it was carried out during the pandemic process. In particular, it is possible to create problems in determining the net effects of remote work on organizational identification levels. Another limitation is the use of the snowball sampling method in data collection. Using this method, the number of participants reached directly and indirectly was limited.

## Author's Note

This study is a part of the doctoral thesis titled “The Antecedents and Consequences of Organizational Role Stress” conducted by AE within the scope of Istanbul Sabahattin Zaim University Graduate Education Institute Business Administration PhD program.

## Data Availability Statement

The original contributions presented in the study are included in the article/supplementary material, further inquiries can be directed to the corresponding author/s.

## Ethics Statement

Ethical review and approval was not required for the study on human participants in accordance with the local legislation and institutional requirements. The patients/participants provided their written informed consent to participate in this study.

## Author Contributions

AE contributed to the research design, data collection, data analysis, and preparation of the manuscript. ÖK provided professional supervision. All authors contributed to the article and approved the submitted version.

## Conflict of Interest

The authors declare that the research was conducted in the absence of any commercial or financial relationships that could be construed as a potential conflict of interest.

## Publisher's Note

All claims expressed in this article are solely those of the authors and do not necessarily represent those of their affiliated organizations, or those of the publisher, the editors and the reviewers. Any product that may be evaluated in this article, or claim that may be made by its manufacturer, is not guaranteed or endorsed by the publisher.
